# Endocarditis with spondylodiscitis: clinical characteristics and prognosis

**DOI:** 10.1186/s12872-021-01991-x

**Published:** 2021-04-15

**Authors:** Stefano Del Pace, Valentina Scheggi, Giacomo Virgili, Sabina Caciolli, Iacopo Olivotto, Nicola Zoppetti, Irene Merilli, Nicole Ceschia, Valentina Andrei, Bruno Alterini, Pier Luigi Stefàno, Niccolò Marchionni

**Affiliations:** 1 Division of General Cardiology, University of Florence, Florence, Italy; 2 Cardiovascular and Perioperative Medicine, University of Florence, Florence, Italy; 3 Institute of Applied Physics “Nello Carrara” (IFAC), National Research Council, Sesto Fiorentino, Italy; 4 Cardiac Surgery, University of Florence, Florence, Italy; 5 Cardiothoracovascular Department, Azienda Ospedaliero-Universitaria Careggi, Florence, Italy; 6 Department of Clinical and Experimental Medicine, University of Florence, Florence, Italy

## Abstract

**Background:**

The association of infective endocarditis (IE) with spondylodiscitis (SD) was first reported in 1965, but few data are available about this issue. This study aimed to evaluate the prevalence of SD in patients with IE, and to determine the clinical features and the prognostic impact of this association.

**Methods:**

We retrospectively analysed 363 consecutive patients admitted to our Department with non-device-related IE. Radiologically confirmed SD was revealed in 29 patients (8%). Long-term follow-up (average: 3 years) was obtained by structured telephone interviews; in 95 cases (13 of whom had been affected by SD), follow-up echocardiographic evaluation was also available.

**Results:**

At univariable analysis, the combination of IE with SD was associated with male gender (*p* = 0.017), diabetes (*p* = 0.028), drug abuse (*p* = 0.009), Streptococcus Viridans (*p* = 0.009) and Enterococcus (*p* = 0.015) infections. At multivariable analysis, all these factors independently correlated with presence of SD in patients with IE. Mortality was similar in patients with and without SD. IE relapses at 3 years were associated with the presence of SD (*p* = 0.003), *Staphylococcus aureus* infection (*p* < 0.001), and drug abuse (*p* < 0.001) but, at multivariable analysis, only drug abuse was an independent predictor of IE relapses (*p* < 0.001; HR 6.8, 95% CI 1.6–29). At echocardiographic follow-up, SD was not associated with worsening left ventricular systolic function or valvular dysfunction.

**Conclusions:**

The association of IE with SD is not rare. Hence, patients with IE should be screened for metastatic infection of the vertebral column, especially if they have risk factors for it. However, SD does not appear to worsen the prognosis of patients with IE, either in-hospital or long-term.

## Background

Despite improved pharmacologic and surgical management, short-term mortality in infective endocarditis (IE) still exceeds 15% [1]. Spondylodiscitis (SD) is a known complication of IE, presumably deriving from haematogenous dissemination of infective agents. The association of IE with SD was first reported in 1965, but this clinical picture has been described only in isolated case reports or clinical series with small numbers [2]. SD is not systematically searched for in patients with IE complaining of back pain and, hence, its prevalence is often underestimated. Moreover, whether the occurrence of SD has a negative prognostic impact on the clinical course of IE, and which are the risk factors and clinical characteristics associated with SD, are issues poorly addressed in previously published clinical series. Therefore, we aimed at evaluating the prevalence of definite SD in patients with IE, identifying any peculiar clinical feature of this association, and assessing the short- and medium-term prognosis of patients with IE complicated with SD.

## Methods

### Patient selection

We retrospectively analysed 363 consecutive patients with non-device-related IE (123 women, 34%; mean age 65.2 ± 14.9 years), admitted to our Department with a definite diagnosis of IE according to modified Duke University criteria, between January 2013 and December 2019. The study was approved by the local ethics Committee that, in keeping with statements by the Italian Regulatory Authorities for retrospective, observational studies (https://www.garanteprivacy.it/web/guest/home/docweb/-/docweb-display/docweb/5805552), granted a waiver of informed consent from study participants. Data for analysis were obtained from electronic hospital charts, anonymized and protected by password. SD was diagnosed by typical clinical signs and symptoms, and confirmed by radiologic findings in all cases.

### Diagnostic work-up

European Society of Cardiology (ESC) guidelines for IE were followed for diagnostic work-up and treatment strategies [3]. In particular, at least three sets of blood cultures were collected and transesophageal echocardiography (TEE) was performed in all patients for diagnosis confirmation. On admission, systemic embolism was sought clinically and radiologically by brain and chest CT plus abdominal CT or ultrasound scan. All patients received appropriate empirical and, when feasible, targeted antimicrobial therapy. Echocardiographic examinations were performed according to the American Society of Echocardiography guidelines [4].

### Surgical indication and operative technique

A Heart Team including a cardiac surgeon, a cardiologist, an anesthesiologist and an internist evaluated each patient. Surgical risk was estimated by Euroscore II [5] and other relevant clinical characteristics. Surgery was advised according to ESC guidelines [3]. Of 363 patients, 77 (21%) were treated conservatively, 39 due to absence of surgical indication and 38 due to prohibitive surgical risk; the remaining 286 (79%) underwent surgical valve repair or replacement.

### Follow-up and study endpoints

Of 363 patients, 29 (8.0%) died in hospital and 334 were discharged, with a 70% [95% CI 65.1–75.3] 3-year survival rate. The duration of follow-up was calculated from the time of IE diagnosis. A structured phone interview was implemented to update the follow-up to December 2019. In a subset of 95 cases, echocardiographic and clinical evaluation was available as well; of these, 13 had had, and 82 had not had SD. Of 334 discharged patients, 77 died during the follow-up and 162 were lost to echocardiographic follow-up and were followed by phone interview only. Our primary objectives were comparing the clinical characteristics and the 30-day and 3-year mortality of patients with and without SD.

### Statistical analysis

The chi-square and the Mann–Whitney or Kruskal–Wallis tests were used to compare respectively proportions and continuous variables with normal or non-normal distribution. Univariable and multivariable analyses were performed using logistic regression and general linear models. The Kaplan–Meier method was used to estimate the survival probability over the follow-up. All tests were 2-sided, and statistical significance was defined as a *p* value < 0.05.

## Results

### Baseline clinical characteristics and microbiology

Valves affected in the 363 cases of IE were aortic in 193 (53%), mitral in 147 (40%) and tricuspid in 23 (7%). Affected valves were native in 231 cases (63%, including eight previous surgical valve repair and one MitraClip), and prosthetic in 132 (37%, including 116 biological surgical prostheses, two TAVI and 14 mechanical prostheses). SD was diagnosed in 29 patients (8%) by clinical criteria (among which, severe back pain in 26, 90%) and confirmatory radiological findings, and was located at the lumbosacral, dorsal and cervical level respectively in 18 (62%), 8 (18%) and in 3 cases (10%).

The main baseline characteristics of the whole series and of patients with and without SD are summarized in Table 1; the underlying valve disease of patients with and without SD is reported in Table 2. Patients with SD were more frequently men, drug abusers and diabetic. When operated, they were more frequently treated with a valve repair than replacement (*p* = 0.007; OR 3.3, 95% CI 1.3–8.4).

**Table 1:** Baseline demographic, clinical, echocardiographic and microbiologic characteristics of 363 patients with IE, by presence of associated spondylodiscitis (SD)

	Total (363)	SD (29)	No SD (334)	*p* value
Age (years), mean ± SD	65.2 ± 14.9	64.6 ± 12.8	65.3 ± 15.1	NS
Gender (male), n (%)	240 (66)	25 (96)	215 (64)	0.017
BMI, mean ± SD	24.9 ± 4.1	25.3 ± 4.0	24.9 ± 4.1	NS
Renal failure, n (%)	96 (26)	5 (17)	91 (27)	NS
Mild n (%)	42 (42)	2 (40)	40 (44)	NS
Moderate n (%)	33 (35)	2 (40)	31 (34)	NS
Severe n (%)	13 (15)	0 (0)	13 (14)	NS
On Dialysis n (%)	8 (8)	1 (20)	7 (8)	NS
Arterial hypertension, n (%)	212 (58)	15 (52)	197 (59)	NS
Previous malignancies, n (%)	80 (22)	6 (20)	74 (22)	NS
Drug abuse, n (%)	43 (12)	8 (27)	35 (10)	0.006
Diabetes, n (%)	68 (19)	10 (34)	58 (17)	0.023
Dyslipidemia, n (%)	110 (33)	11 (37)	99 (29)	NS
Pacemaker, n (%)	42 (12)	4 (14)	38 (12)	NS
Prosthetic valve, n (%)	132 (38)	8 (28)	124 (37)	NS
First episode of IE, n (%)	330 (91)	26 (90)	304 (91)	NS
Euroscore 2, mean ± SD	12.4 ± 16.5	8.5 ± 7.4	12.7 ± 17.0	NS
Systemic embolism, n (%)	129 (35)	12 (41)	117 (35)	NS
Cerebral embolism	74 (51)	3 (10)	71 (21)	NS
Retinal embolism	4 (1)	0 (0)	4 (1)	NS
Coronary embolism	2 (1)	0 (0)	2 (1)	NS
Extremities embolism	11 (3)	1 (3)	10 (3)	NS
Abdominal embolism	40 (11)	4 (14)	36 (11)	NS
Pulmonary embolism, n (%)	20 (5)	5 (17)	15 (5)	0.015
Perivalvular extension, n (%)	84 (23)	3 (10)	81 (24)	NS
Severe valvular dysfunction, n (%)	172 (47)	16 (55)	156 (47)	NS
Vegetation length (mm), mean ± SD	9.1 ± 7.6	10.4 ± 5.6	9.0 ± 7.8	NS
Left Ventricular Ejection Fraction (%), mean ± SD	56.7 ± 9.9	58.2 ± 7.4	56.6 ± 10.1	NS
TAPSE (mm), mean ± SD	20.8 ± 5.6	21.7 ± 6.4	20.7 ± 5.5	NS
Valve repair in surgical patients, n/tot (%)	40/286 (14)	8/25 (32)	32/261 (12)	0.007
Streptococcus Viridans, n (%)	62 (16)	10 (35)	52 (16)	0.009
*Streptococcus bovis*, n (%)	25 (7)	1 (3)	24 (7)	NS
*Staphylococcus aureus*, n (%)	64 (18)	3 (10)	61 (18)	NS
Negative coagulase Staphylococci, n (%)	46 (13)	1 (3)	45 (13)	NS
Enterococci, n (%)	65 (18)	10 (35)	55 (16)	0.015
Other, n (%)	27 (7)	2 (7)	25 (8)	NS
Negative culture, n (%)	74 (20)	2 (7)	72 (22)	NS

*BMI* body mass index. Renal failure: GFR < 60 mL/min/1.73 m^2^ (Mild, GFR 45–59; Moderate, GFR 30–44; Severe, GFR 15–29); *TAPSE* tricuspid annular plane excursion

**Table 2:** Underlying valve disease in patients with and without spondylodiscitis

Type of valve	Site	Spondylodiscitis					
		Yes			No		
		Valvula dysfunction n (%)			Valvula dysfunction n (%)		
		Absent	Mild/moderate	Severe	Absent	Mild/moderate	Severe
Native	Aortic	1 (100)	1 (20)	5 (33)	16 (57)	29 (45)	54 (46)
	Mitral	0 (0)	3 (60)	8 (54)	10 (36)	31 (47)	56 (48)
	Tricuspid	0 (0)	1 (20)	2 (13)	2 (7)	5 (8)	7 (6)
Prosthetic	Aortic	4 (100)	2 (67)	1 (100)	19 (70)	36 (62)	25 (64)
	Mitral	0 (0)	0 (0)	0 (0)	8 (30)	19 (33)	12 (31)
	Tricuspid	0 (0)	1 (33)	0 (0)	0 (0)	3 (5)	2 (5)

Blood cultures were positive in 80% of cases. Microbiological cultures were similar in patients with and without SD, with the only exception of Streptococcus Viridans and Enterococci, which were both more frequent in those with SD (Table 1). Notably, Staphylococcus was found more frequently in drug-addicted than in non-addicted patients (25/43; 58%, vs. 85/320, 26% *p* < 0.001). In our cohort, Staphylococcus was not associated with the combination of IE and SD. Conversely, the prevalence of Streptococcus Viridans and Enterococcus infection was similar in drug- and not drug-addicted patients. These observations confirm that drug addiction and Enterococcus are independent risk factors for SD. Embolic events other than SD, such as in particular brain embolism, were present on admission in similar proportions in patients with and without SD. Only pulmonary embolism was more frequent in patients with SD, likely because of the high proportion of drug abusers. At multivariable analysis, male gender, diabetes, drug abuse, and infection from Streptococcus Viridans or Enterococcus were all factors significantly associated with the presence of SD (Table 3).

**Table 3:** Multivariable analysis of factors associated with spondylodiscitis

	OR	95% CI		*p* value
Male gender	3.60	1.16	11.14	0.026
Drug abuse	6.02	2.11	17.13	0.001
Diabetes	3.74	1.45	9.67	0.006
Streptococcus Viridans	7.08	2.51	19.92	0.000
Enterococcus	4.42	1.58	12.30	0.004

### In-hospital and long-term clinical outcomes

**Fig. 1:**
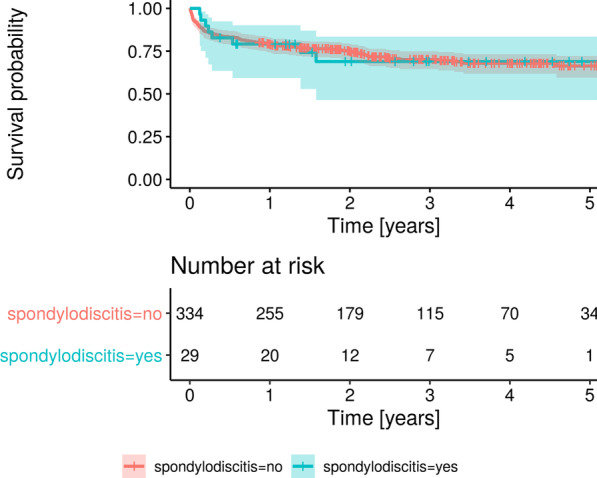
Kaplan–Meier analysis of survival probability in patients with (YES) and without (NO) spondylodiscitis. The shaded areas depict 95% CI

Duration of hospital stay averaged 10 days in both SD and no-SD groups (Table 3). IE relapse within 3 years was associated with SD (*p* = 0.003), but also with *Staphylococcus aureus* infection (*p* = 0.004), and drug abuse (*p* < 0.001). However, at multivariable analysis only drug abuse was retained as an independent predictor of IE relapses (*p* = 0.009; HR 6.8, 95% CI 1.6–29). At echocardiographic follow-up, SD was not associated with worsening left ventricular ejection fraction or valvular dysfunction (data not shown). Mortality was similar in patients with and without SD both at 30 days and 3 years since first diagnosis (Table 4), and survival curves at Kaplan–Meier analysis were also similar over the whole follow-up (Fig. 1).

**Table 4:** Short- and long-term outcomes of 363 patients with IE, by presence of associated spondylodiscitis (SD)

	Total (363)	SD (29)	No SD (334)	*p* value
Length of hospital stay (days), mean ± SD	10.2 ± 18.1	11.4 ± 17.8	10.1 ± 18.2	NS
All-cause death at 30 days, n (%)	33 (9)	0 (0)	33 (10)	NS
Relapse at 3 years, n (%)	19 (5.2)	5 (17.2)	14 (4.2)	0.003
All-cause death at 3 years, n (%)	106 (29)	8 (27)	98 (29)	NS

## Discussion

In our retrospective analysis of 363 consecutive patients with IE, the prevalence of associated SD was 8.0% (n = 29). In a smaller series of 58 patients with IE [6], the reported prevalence of vertebral osteomyelitis was even higher (19%): beyond the much smaller number of IE in that clinical series, this difference might be due to the strict criteria adopted in our study, where we included only SD cases with radiologically proven findings coherent with the clinical suspicion. Since 1965, when the association of IE with SD was first reported [1], the frequency of this clinical picture seems to increase [6]. This observation may depend on the improvement of diagnostic tools, such as bone CT, MRI and PET, that are of great aid in the differential diagnosis with rheumatologic disorders [7]. Similarly, therapeutic options for SD have grown over time, improving its prognosis. Prolonged antibiotic therapy is the mainstay of SD treatment, reserving surgery for complicated cases [7]. Still, the clinical features and the prognostic impact of the association of SD with IE is a matter of debate.

The first query we answered in our study was the identification of the clinical features characterizing patients with SD complicating an IE. We found independent associations of this picture with male gender, intravenous drug abuse, diabetes and infection by Streptococcus Viridans or Enterococcus. The main novelty of the present study is that the combination of SD and IE is associated with intravenous drug abuse and Enterococcus infection, as male gender and diabetes already had been reported as independent risk factors for such clinical combination [2]. On the other hand, intravenous drug abuse is a well-known condition predisposing to isolated SD through haematogenous dissemination [7]. This observation, and the unusually high rate of left-sided IE in intravenous drug abusers (62%), might support the hypothesis that, in this population, SD is the initial infective focus, which subsequently spreads to cardiac valves.

Since most of our patients had severe back pain at presentation, a history of intravenous drug abuse associated with back pain should prompt further diagnostic workup to exclude spinal infection and, in such conditions, IE should be systematically ruled out, even in the absence of other signs of systemic infection. Indeed, an undetected IE in a patient undergoing spine surgery might compromise the success of surgical treatment and the overall prognosis.

In patients with IE, concomitant SD has been found most often associated with Staphylococci and Streptococci infections [7, 8] and only seldom with Enterococci [6, 9, 10], while our data support the view of a substantial incidence of Enterococci as etiologic agents of IE and concomitant SD. Since isolated SD is not a typical consequence of Enterococcus infection, in patients with IE it is reasonable to hypothesize an embolic pathogenesis. For this reason, SD should be accurately searched for in any patient with Enterococcus IE complaining of back pain.

The second research query we answered, was the prognostic impact of SD combined with an IE. Consistently with previously reported data based on much smaller series [2, 6], the presence of SD did not result to affect either the short- or the long-term prognosis of our IE patients. We found significantly more relapses in patients with SD, likely attributable to the relevant proportion of drug abusers in this subset. In our experience, SD was not associated with either worsening LV systolic function or valvular dysfunction as assessed in the subset of patients with echocardiographic follow-up.

With regard to surgical treatment, we found a remarkably higher proportion of valves that could be repaired rather than replaced, previously unreported in this clinical context. Valve repair should be preferred over replacement whenever possible, not only in patients with degenerative disease but also in those with IE, since it is safe, durable and, according to some published series [11, 12], associated with a lower incidence of relapses. We believe that the presence of SD, which may act as a persisting infective focus, should be considered as one further reason to attempt at valve repair in IE whenever anatomically feasible, in order to possibly reduce the risk of early relapse.

### Study limitations

The main study limitation is its retrospective nature, based on a single center experience, thereby including a relatively small sample size. However, to the best of our knowledge, our population of SD during IE is the largest one ever published on this issue, covering a long study period (maximum follow-up 6 years) and with a 3-year average follow-up. Moreover, given the retrospective analysis, we were able to exclude from the study the cases of SD not fulfilling all the clinical and radiological diagnostic criteria, thereby avoiding to overestimate its real prevalence. On the other hand, prospective studies would be useful and perhaps more accurate, but they are hardly feasible, due to the low incidence of the association. Finally, our study has a potential referral bias, since it was conducted in a high-volume surgical center, thereby limiting the referral of uncomplicated IE eligible to medical therapy.

## Conclusions

In our cohort, patients with SD were a relatively considerable proportion (8%) of all cases admitted for IE. Therefore, this potential association must be kept in mind during the clinical evaluation of IE. Indeed, an undetected SD might lead to an inadequate duration of antibiotic therapy (6 weeks rather than 3 months, as recommended by current guidelines) of IE. Some factors in particular should raise the clinical suspicion of SD, i.e. male gender, diabetes, history of drug abuse and Enterococcal infection. Relapse rate was higher in those with SD but this was likely due to the high proportion of drug-addicted patients. Overall, we observed no difference in short- or long-term mortality, worsening LV systolic dysfunction, or valvular dysfunction at follow-up between patients with and without SD.

## Data Availability

The datasets used and/or analysed during the current study are available from the corresponding author on reasonable request.
